# Short-Term Clinical Outcomes after Using Novel Deeper Intubation Technique (DIT) of Ileus Tube for Acute Bowel Obstruction Patients

**DOI:** 10.1155/2020/1625154

**Published:** 2020-05-15

**Authors:** Yanlu Tan, Haibin Chen, Wenji Mao, Qin Yuan, Jun Niu

**Affiliations:** ^1^Department of Interventional Surgery, Central Hospital of Zibo, Zibo, China; ^2^Department of General Surgery, The First Affiliated Hospital of Nanjing Medical University, Nanjing, China; ^3^Department of Radiology, Central Hospital of Zibo, Zibo, China; ^4^Outpatient Department, Central Hospital of Zibo, Zibo, China; ^5^Department of General Surgery, Qilu Hospital of Shandong University, Jinan, China

## Abstract

**Background:**

The ileus tube has been widely used for the treatment of acute small bowel obstruction. However, it is difficult to get the tube sufficiently adjacent to the obstruction site due to various reasons.

**Methods:**

We developed a novel intubation technique, named Deeper Intubation Technique (DIT), by using the Zebra Urological Guidewire and digital gastrointestinal fluoroscopy, where we deepened the catheter intubation, and further compared the effects of DIT with the Traditional Intubation Technique (TIT) on the short-term clinical outcomes of 183 patients.

**Results:**

The average intubation depth of DIT apparently exceeds that of TIT (213.89 ± 31.11 vs. 134.67 ± 18.22 cm, *P* < 0.001). Compared with patients in the TIT group, patients in the DIT group got a lower pain score (*P* < 0.001), shorter recovery time for anal exhaust defecation (2.87 ± 1.50 vs. 3.37 ± 1.52 d, *P* = 0.040), higher recovery rate in anal exhaust defecation (24 h, 16.8% vs. 5.7%, *P* = 0.021; 48 h, 46.3% vs. 27.3%, *P* = 0.009), better symptomatic remission rate and imaging relief rate (*P* < 0.05), and increased drainage volume (1006.88 ± 583.45 vs. 821.02 ± 358.73 ml, *P* = 0.009). Importantly, the emergency surgery rate in the DIT group was lower than that in the TIT group (3.2% vs. 13.6%, *P* = 0.014). In addition, the DIT procedure was effective for patients with adhesive obstruction but not for cancerous and stercoral bowel obstruction.

**Conclusion:**

Compared to TIT, DIT produced better short-term clinical outcomes, indicating that DIT is a safe and feasible technique for the treatment of adhesive intestinal obstruction.

## 1. Introduction

Intestinal obstruction is one of the most common reasons of all emergency department visits for acute abdominal pain [1]. Among all the conventional methods to treat the simple intestinal obstruction such as gastrointestinal decompression, fasting, pain control, intravenous fluid replacement, correction of electrolyte imbalances, and anti-infection, gastrointestinal decompression appears to be the most important [2]. Originally, the nasogastric tube (Levin tube) decompression was widely used. In 1953, a Japanese scholar officially presented a novel treatment strategy for intestinal obstruction named the ileus tube. The effect of this treatment was gradually confirmed and used [3–7]. With guidance of X-ray or electronic gastrointestinal endoscopy, the tube is usually placed in the jejunum with its tip through the pylorus, duodenum, and the ligament of Treitz. However, due to deficiencies of conventional ileus tube intubation such as high friction between tubes and guide wires, consistently shifting position, and the resistance of the intestine inner fold, it is difficult to get the tube sufficiently adjacent to the obstruction site by merely incubating the duodenum or the ligament of Treitz, with the intubation depth not more than 150 cm.

In the present study, we proposed a novel intubation method that allows the tube placement into the proximal end of the obstruction via the digital gastrointestinal fluoroscopy and Zebra Urological Guidewire, which was named the Deeper Intubation Technique (DIT). To evaluate the safety and feasibility of DIT, we retrospectively compared the short-term clinical outcomes of patients treated with DIT and of the Traditional Intubation Technique (TIT) during the same period.

## 2. Materials and Methods

### 2.1. Patients

This retrospective study investigated 183 hospitalized patients with acute intestinal obstruction from January 2014 to December 2017 in the Central Hospital of Zibo. The criteria for case selection were listed as follows: (1) hospitalized patients had acute intestinal obstruction symptoms like nausea and vomiting, abdominal pain, abdominal distension, and exhaust defecation ceasing; (2) patients diagnosed with intestinal obstruction by abdominal X-ray plain films and abdominal CT examination; (3) patients suitable for conservative treatment with no severe abdominal pain or persistent abdominal pain, bloody vomiting or bloody stool, asymmetric abdominal distension, respiratory instability and even shock, peritoneal irritation, and other strangulated intestinal obstruction symptoms; (4) patients had no contraindications of tube intubation, such as the history of ENT surgery, and esophageal disease; and (5) detailed medical records and follow-up information were available.

In the DIT group, 95 patients (48 males and 47 females) aged 45-96 years old (63.7 years old on average) were treated with the Deeper Intubation Technique, among whom 59 patients had a history of abdominal operations. While in the TIT group, 88 patients were treated with traditional techniques, 47 males and 41 females aged 26-88 years old (61.6 years old on average), among whom 57 had the history abdominal surgery.

All the experimental protocols were approved by the Ethics Committee of Central Hospital of Zibo, China. Written informed consent was obtained from all subjects.

### 2.2. Methods

#### 2.2.1. Instrument Preparation

**Figure 1:**
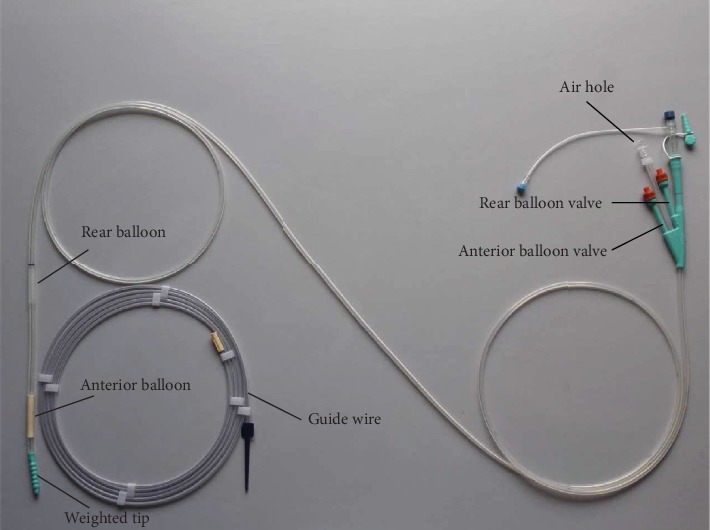
The schematic diagram of the CLINY Ileus Tube suite (Create Medic, Tokyo, Japan). The tube is 300 cm in length, 16 Fr or 18 Fr. The front of the tube is composed with a weighted tip, anterior balloon, and rear balloon. At the end of the tube, there is an anterior balloon valve (marked F. BALL), an air hole (marked VENT), and a rear balloon valve (marked B. BALL).

Both groups adopted the CLINY Ileus Tube suite (Create Medic, Tokyo, Japan) (Figure 1). The ileus tube is 300 cm in length, whose commonly used specifications are 16 Fr and 18 Fr with three channels and two balloons. The facade of the tube has a weighted tip that is composed of six successive steel balls (no penetration of X-ray), which was used to facilitate the tube through the pylorus and guide the tube forward. Distilled water could be injected into the anterior balloon to advance the tube as well as using the rear balloon in high selective intestinal radiography. At the end of the tube, there are an anterior balloon valve, air hole, and rear balloon valve.

Digital gastrointestinal fluoroscopy (TU-51, Hitachi) and a Zebra guide wire (Nanjing Micro-Tech Co., Ltd., 4500 mm, *Φ*0.035 inches) were adopted to complete the DIT.

A traditional technique-approved DSA operating instrument (Artis Zeego, Siemens Healthineers) and the guide wire were matched with the CLINY Ileus Tube suite with the length of 3.5 meters and 4.5 meters, *Φ*0.045 inches.

#### 2.2.2. Intubation Methods

*(1) Deeper Intubation Technique*. The patient was locally anesthetized, and the tube was placed in the stomach through the nose. Then, the position of the patient was transformed, and the tube was observed by gastrointestinal fluoroscopy. We place the tip of the tube near the stomach, pylorus, and duodenal suspensory ligament, with 60 cm, 85 cm, and 110 cm marked. (For patients with digestive tract reconstruction, it is necessary to decide the intermittent length according to the reconstruction.) When the tube was blocked or coiled, it would demand to rotate the tube, change the patient's posture, adjust the guide wire, and continue to push the tube. As the tube reached the jejunum, we observed the tube position every 15 cm pressed, where the Zebra guide wire guides the tube while draining intestinal contents at the same time, placing the tube close to the obstruction site. In the intubation process, we need to constantly change the patient's position and facilitate the tube intubated downwards. If the tube went downwards with difficulties, appropriate air volume could be injected into the intestine via the tube to change the position of small bowels and then repeat the manipulation. It was also useful to pump water-soluble iodine contrast medium, which can be applied to observe the direction of bowel movement, stimulate the peristalsis, reduce bowel wall edema, and increase the secretion of digestive juices, so that we can push the tube downwards easily [8–10]. After the operation completion, 15 ml of sterile water would be injected into the anterior balloon connecting the end bore with a little negative pressure drainage (980-2450 Pa or 10-25 cm water column). We fixed the exposed tube on the cheek beside the nose and reviewed the abdominal plain film every 24 hours. Based on the patient's clinical symptoms, bowel dilatation, and tube position, we can decide to push the tube to further adjust the size of the anterior balloon or perform the abdominal radiography.

*(2) Traditional Intubation Technique*. It is required that DSA fluoroscopy and the guide wire match with the CLINY Ileus Tube suite. The tube would approach the stomach of the patient through the nose after local anesthetization. Under X-ray, the front of the tube reached the large curvature of the stomach while patients remained at the left anterior oblique position. Then, patients should be turned to a left lateral position to keep the head of the tube toward the pylorus, and the guide wire would be inserted first to pass the pylorus where the tube would get directed into the duodenum aided by the guide wire. Once the tube reached the jejunum through the ligament of Treitz, the friction between tubes and guide wires might enlarge. The friction would lead to loss of control of the tube, and it becomes difficult to draw the guide wire back. The following steps after the completion of intubation were the same as the DIT.

All patients were given such conservative treatment methods as fasting, intravenous nutrition, anti-infection, maintenance of water, electrolyte and acid-base balance, etc. When patients presented with symptoms of severe abdominal pain, worsened distension, tachycardia, hematemesis, hematochezia, peritoneal irritation, isolated swelling bowel loops, and even shock, timely surgical treatment would be needed.

### 2.3. Outcome Measurement

Patients' pain score and defecation situation should be monitored and recorded before and after the intubation every 24 hours with the patients' pain-scoring Numeric Rating Scale (NRS). The abdominal position flat film or CT would be reviewed every 24 hours in three days after intubation, and the number of gas-liquid levels and the degree of intestinal dilatation would be recorded while the daily drainage of the gastrointestinal decompression tube would be documented.

The average intubation depth, the volume of drainage, the pain score, the abdominal pain relief rate, and the recovery time for anal exhaust defecation were recorded. The treatment efficiency was defined as a clinical or radiological improvement, relief of abdominal symptoms, decreased drainage volume, and disappearance of air-fluid levels [11, 12].

### 2.4. Statistical Analysis

SPSS 20.0 was used for the statistical analysis. Comparisons between the two groups were performed by Student's *t*-test for continuous variables and the chi-square test or Fisher's exact test for categorical variables. All statistical tests were two-sided, and *P* values less than 0.05 were considered significant.

## 3. Results

### 3.1. Clinical Characteristics of Patients

All patients had developed acute intestinal obstruction symptoms such as abdominal pain, abdominal distension, nausea, vomiting, and failure of stool and gas pass and had no symptoms suggesting strangulated intestinal obstruction, which proved the requirements of conservative treatment. There were no significant differences in average age and sex ratio between the two groups (*P* > 0.05). According to medical history, physical examination, and imaging examination, patients were divided into four types including malignant (cancerous) obstruction, adhesions, and fecal obstruction. As shown in Table 1, the baseline clinical characteristics of the patients between the two groups were of no statistic difference.

### 3.2. Short-Term Outcomes after DIT or TIT Treatment

**Figure 2:**
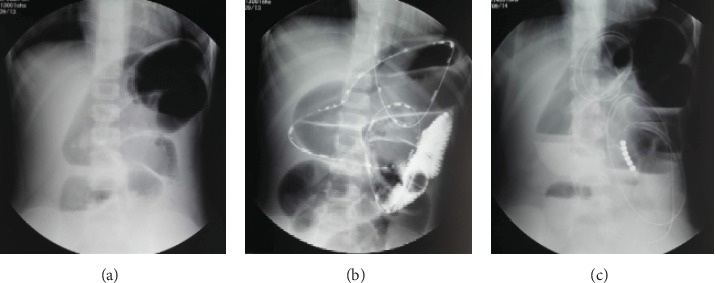
Presentative abdominal X-ray plain films of the TIT procedure. (a) Before intubation, the proximal intestinal canal dilated obviously according to the plain films. (b) The patient was treated with the TIT procedure, and the intubation depth was 135 cm. (c) 24 hours after intubation, the dilatation of intestine was relieved.

**Figure 3:**
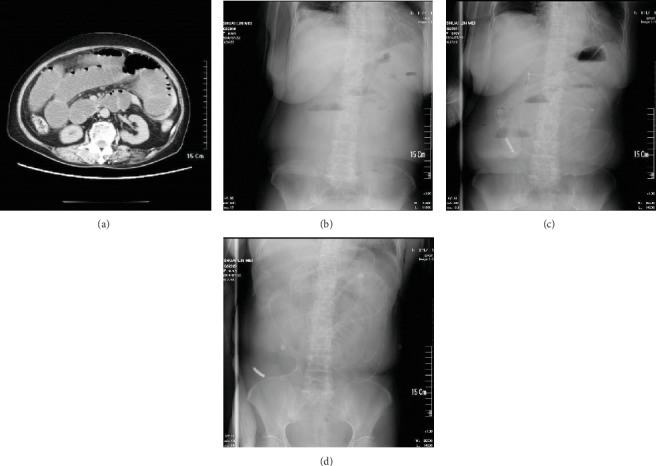
Presentative abdominal X-ray plain films of DIT procedure. (a, b) Before intubation, there were lots of gas-liquid levels, and the proximal intestinal canal dilated obviously. (c) The patient was treated with the DIT procedure, and the intubation depth was up to 265 cm. (d) 24 hours after intubation, the gas-liquid levels had almost disappeared.

As shown in Figure 2, the TIT treatment could reach the proximal jejunum. While for the DIT group, the intubation could obtain the ileum of the patients with a maximum depth up to 265 cm (Figure 3).

Table 2 provides the detailed short-term outcomes between the DIT and the TIT group. We could see that the intubation depth in the DIT group was significantly deeper than that in the TIT group (213.89 ± 31.11 vs. 134.67 ± 18.22 cm, *P* < 0.001).

No significant difference in NRS scores was shown between the two groups before intubating the ileus tube (5.39 ± 1.22 vs. 5.18 ± 1.14, *P* = 0.237), while the NRS score in the DIT group was significantly lower than that in the TIT group 24 (4.26 ± 1.15 vs. 4.76 ± 1.10, *P* = 0.003) and 48 hours (3.25 ± 0.99 vs. 3.86 ± 1.12, *P* < 0.001) after intubation.

With regard to defecation, we observed that the exhaust defecation time was significantly shortened in the DIT group compared to the TIT group (2.87 ± 1.50 vs. 3.37 ± 1.52 days, *P* = 0.040). The defecation rate within 24 (16.8% vs. 5.7%, *P* = 0.021) and 48 hours (46.3% vs. 27.3%, *P* = 0.009) after intubation was markedly increased in the DIT group. More than half of the patients in both groups recovered exhaust defecation within 72 hours (61.1% vs. 51.1%, *P* = 0.184). While 7 days after incubation, most of the patients in both groups showed recovered defecation (90.5% vs. 83%, *P* = 0.188).

With respect to remission of the disease, we observed the relief condition via two parameters, symptomatic remission and imaging relief. Time for symptomatic remission in the DIT group was 2.15 ± 1.33 days, compared to 2.79 ± 1.35 days in the TIT group (*P* = 0.005). The symptomatic remission rates in 24 hours, 48 hours, and 72 hours were 25.5%, 63.2%, and 86.3% in the DIT group, respectively. While in the TIT group, the symptomatic remission rates in 24 hours, 48 hours, and 72 hours were 9.1%, 38.6%, and 68.2%, respectively. Abdominal X-ray plain film was conducted daily after intubation, and the situation of patients' bowel dilatation and gas-liquid levels was recorded. As for imaging relief condition, the time for imaging relief was 2.40 ± 1.34 days in the DIT group and 3.00 ± 1.23 days in the TIT group (*P* = 0.008). In the DIT group, the imaging relief rates in 24 hours, 48 hours, and 72 hours were 21.1%, 54.7%, and 82.1%, respectively, whereas those in the TIT group were 8.0%, 30.7%, and 62.5%, respectively.

The drainage volume of the DIT group in the first 24 hours after intubation was significantly higher than that of the TIT group (1006.3 ± 583.45 ml vs. 821.02 ± 358.73 ml, *P* = 0.09). Although there was no significant difference in the surgery rate between the two groups, the DIT group showed a lower tendency for surgery compared to the TIT group (12.6% vs. 19.3%, *P* = 0.066). Moreover, the emergency surgery rate was significantly lower in the DIT group compared to the TIT group (3.2% vs. 13.6%, *P* = 0.014).

### 3.3. The Overall Efficacy for Different Types of Intestinal Obstruction

As shown in Table 3, 56 patients were diagnosed with adhesive intestinal obstruction, and 50 (89.3%) of them recovered after intubation in the DIT group. While in the TIT group, a total of 52 patients were diagnosed with adhesive intestinal obstruction, and 38 (73.1%) patients showed adequate curing (*P* = 0.030). No significant difference was shown between the two groups when it comes to fecal obstruction (*P* = 0.630) and cancerous obstruction (*P* = 0.445).

## 4. Discussion

For patients with acute intestinal obstruction, gastrointestinal decompression can drain intestinal contents from proximal obstruction, reduce the pressure of the duodenal cavity, and restore duodenal blood circulation, which could improve patients' symptoms, avoid intestinal necrosis, and decrease intestinal bacterial translocation. Research has demonstrated that the decompression effect of intestinal obstruction is significantly better than the traditional nasogastric tube [12–14]. It has been reported that the decompression efficiency of the traditional nasogastric tube only lies in 30-40% [15–17], while the effectiveness of the ileus tube was increased to 70-80% [12, 18]. Therefore, a number of scholars suggested that the ileus tube, instead of the nasogastric tube or surgery, should be the first choice for patients with acute intestinal obstruction, after excluding strangulation obstruction or other contraindications [3, 17, 19]. However, the nasogastric tube is still the preferred treatment due to its simple operation in clinical practice.

Commonly, intubation methods of the ileus tube could be classified into X-ray-guided intubation and endoscopy-guided intubation. The traditional intubation method is inserting the tip of the tube through the ligament of Treitz and filling the front balloon so that the tube could move forward under the drive of intestinal peristalsis. Once the tip of the tube passes through the ligament of Treitz, operation will become difficult, which may result from several reasons. First of all, the guide wire matching the CLINY Ileus Tube suite has a diameter of 0.045 inches and a high degree of hardness. The friction between the guide wire and tube will be increased with the increase of intubation depth, resulting in considerable difficulties in controlling the tube and guide wire and even failing to pull out the guide wire [20]. Secondly, during the operation, patients' position needs to be constantly changed in order to facilitate the tube intubation. Patients with intestinal obstruction are poorly tolerated and unable to cope with postural changes. Thirdly, intestinal folds may serve as obstacles for forwarding the tube.

Based on the aforementioned problems, we improved the traditional technique as follows, which is named DIT. Firstly, when the tip of the tube gets into the jejunum, intubation should be continued to move forward the tip as far as possible. Then, to reduce the friction between the guide wire and tube, a finer and softer zebra guide wire with the diameter of 0.035 inches is selected. With the help of the Zebra guide wire, it will be easier to control the tube when the intubation depth is increased. Moreover, as an operating platform for intubation of the ileus tube, digital gastrointestinal fluoroscopy is easier for changing patients' posture than DSA. Another important advantage is that doctors can operate intermittently with digital gastrointestinal fluoroscopy, avoiding continuous X-ray radiation of DSA. When intubation encounters obstacles such as intestinal folds or swerve, an appropriate volume of air could be injected into the bowel via the catheter to change the stereo direction of the bowel, changing patients' posture and guiding the weighted tip to conform to the track of the bowel at the same time. In addition, operators can inject the water-soluble iodine contrast medium into the bowl to observe the stereo track of the bowel and stimulate peristalsis [8–10]. With the DIT procedure, the tip of the tube can reach the obstruction more closely and decompress the intestine contents more sufficiently, which effectively reduces bowel dilatation and promotes the recovery of bowel function [14].

The two groups were comparable as no significant difference was shown in age, sex ratio, previous laparotomies, and obstruction types, etc. In the DIT group, the mean intubation depth is up to 213.89 cm, obviously greater than that of the TIT group. Intestinal peristalsis is the major power promoting the tube forward in TIT. However, due to the application of analgesics, abdominal (intestinal) infection, and electrolyte disorders, patients usually present with weakened or even vanished intestinal peristalsis. This is one of the key reasons that decrease the effectiveness of the TIT procedure. By DIT, operators can easily intubate the tip of the tube to the distal end of the jejunum; even in the condition where the intestinal peristalsis is weakened, the satisfactory drainage could also be obtained. The ileus tube quickly drains the effusion and pneumatosis in the bowel and facilitates the recovery of intestinal blood supply. As bowel diameter decreases and intestinal peristalsis recovers, the tube will be pushed forward, leading to a positive feedback. So patients' symptoms in the DIT group could be relieved faster.

The present study results have shown the advantages of DIT in intestinal decompression, with significantly increased drainage in 24 hours. Meanwhile, patients' abdominal pain was relieved faster, and patients in the DIT group recovered faster, both symptomatically and radiologically. As time went on, the ileus tube would move towards the obstruction due to the gravity action of the tip of the tube and the intestinal peristalsis. As the tube moved forward, the tip of the tube in the TIT group could reach the same site with the DIT group finally. Therefore, the advantage of DIT in the early stage of acute intestinal obstruction is more evident. This may account for the lower emergency surgery rate in the DIT group. Reducing the emergency surgery rate is of vital importance for the treatment of intestinal obstruction. The surgeons could make a sufficient preoperative preparation improving patients' general situation, which can reduce complications and mortality. In addition, the DIT procedure could improve patients' symptoms rapidly and convert emergency surgery into nonemergency surgery. All the aforementioned effects of the DIT procedure led to better short-term outcomes for patients with intestinal obstruction.

The effect of DIT on different types of intestinal obstruction was also investigated. For adhesive intestinal obstruction, the most common type of intestinal obstruction [11, 21], the overall efficacy for the DIT procedure was up to 89.3%. Some patients suffering from recurrent attacks of intestinal obstruction usually have severe ankylenteron and have undergone abdominal operations several times. For those patients, surgery is quite challenging and risky and the complication rate is high. Besides, surgery treatment may aggravate the ankylenteron and induce the recurrence of intestinal obstruction [22]. The DIT procedure could increase the efficacy of conservative therapy and decrease the operation rate, which could help patients avoid surgery and reduce the economic burden. In clinical practices, the tip of the tube will reach the obstruction site more quickly using the DIT procedure, and dissolution therapy can be performed earlier than the TIT procedure. For patients with fecal obstruction, the aerogenic agent (mainly composed of citric acid and sodium bicarbonate) or liquid paraffin would be given through the tube, which could facilitate the dissolution and cure of stercolith. There was no significant difference in the efficacy of the two methods for patients with malignant obstruction, and the efficacy is the worst among the three types of intestinal obstruction, which was consistent with previous studies [14].

One of the important limitations of the present study is the lack of long-term survival. Further study investigating the prognosis of patients in the DIT group is warranted. Moreover, as the baseline parameters of patients of the two groups were similar (number of patients, age, sex, obstruction type, and history of abdominal surgery) and the number of patients is limited, case-matched analysis was not available in the present study. In addition, multicenter comparative prospective studies with increased number of cases are needed to verify the safety and effectiveness of DIT.

## 5. Conclusion

In conclusion, this study presented a novel technique which could significantly improve the short-term clinical outcomes of intestinal obstructive patients, especially for the patients with adhesive obstruction. The DIT may serve as a safe and effective procedure for the patients with intestinal obstruction.

## Figures and Tables

**Table 1:** Baseline clinical characteristics of patients between the two groups.

Clinical characteristics	DIT group	TIT group	*P* value
Number	95	88	—
Mean age (year)	63.7 ± 14.9	61.6 ± 16.1	0.261
Sex ratio (male/female)	48/47	47/41	0.697
Intestinal obstruction type			
Adhesive obstruction	56	52	0.984
Cancerous obstruction	12	9	0.610
Fecal obstruction	13	11	0.813
Other types	14	16	0.529
History of laparotomies/none	64/31	62/26	0.750
Surgery type			
Hysterectomy, adnexectomy, or hysterectomy	17	19	0.530
Subtotal gastrectomy	18	9	0.530
Colorectal surgery	10	16	0.138
Appendectomy	6	5	0.857
Operation on intestine	5	4	0.823
Cholecystectomy	3	4	0.735
Herniorrhaphy	2	1	—
Cystectomy	1	0	—
Splenectomy	1	1	—
Nephrectomy	1	1	—
Hepatic carcinectomy	0	2	—

**Table 2:** Short-term outcomes of DIT group and TIT group.

Outcome measures	DIT group	TIT group	*P* value
Intubation depth (cm)	213.89 ± 31.11	134.67 ± 18.22	<0.001^∗^
NRS score before intubation	5.39 ± 1.22	5.18 ± 1.14	0.237
NRS score after 24 hours	4.26 ± 1.15	4.76 ± 1.10	0.003^∗^
NRS score after 48 hours	3.25 ± 0.99	3.86 ± 1.12	<0.001^∗^
Exhaust defecation time (day)	2.87 ± 1.50	3.37 ± 1.52	0.040^∗^
Defecation recovery rate in 24 hours	16.8% (16/95)	5.7% (5/88)	0.021^∗^
Defecation recovery rate in 48 hours	46.3% (44/95)	27.3% (24/88)	0.009^∗^
Defecation recovery rate in 72 hours	61.1% (58/95)	51.1% (45/88)	0.184
Defecation recovery rate 7 days later	90.5% (86/95)	83.0% (73/88)	0.188
Time for symptomatic remission (days)	2.15 ± 1.33	2.79 ± 1.35	0.005^∗^
Symptomatic remission rate in 24 hours	25.3% (24/95)	9.1% (8/88)	0.006^∗^
Symptomatic remission rate in 48 hours	63.2% (60/95)	38.6% (34/88)	0.001^∗^
Symptomatic remission rate in 72 hours	86.3% (82/95)	68.2% (60/88)	0.004^∗^
Time for imaging relief (day)	2.40 ± 1.34	3.00 ± 1.23	0.008^∗^
Imaging relief rate in 24 hours	21.1% (20/95)	8.0% (7/88)	0.021^∗^
Imaging relief rate in 48 hours	54.7% (52/95)	30.7% (27/88)	0.002^∗^
Imaging relief rate in 72 hours	82.1% (78/95)	62.5% (55/88)	0.005^∗^
Drainage in 24 hours (ml)	1006.88 ± 583.45	821.02 ± 358.73	0.009^∗^
Surgery rate	12.6% (12/95)	19.3% (17/71)	0.066
Emergency surgery rate	3.2% (3/95)	13.6% (12/88)	0.014^∗^

^∗^ *P* < 0.05.

**Table 3:** Therapeutic efficacies for different types of intestinal obstruction.

Overall efficacy (effective/ineffective)	DIT group	TIT group	*P* value
Adhesive obstruction	89.3% (50/6)	73.1% (38/14)	0.030∗
Fecal obstruction	84.6% (11/2)	72.7% (8/3)	0.630
Cancerous obstruction	50.0% (6/6)	66.7% (6/3)	0.445

^∗^ *P* < 0.05.

## Data Availability

The original data used to support the findings of this study are available from the corresponding author upon request.
